# A framework for implementation, education, research and clinical use of ultrasound in emergency departments by the Danish Society for Emergency Medicine

**DOI:** 10.1186/1757-7241-22-25

**Published:** 2014-04-15

**Authors:** Christian B Laursen, Klaus Nielsen, Minna Riishede, Gerhard Tiwald, Anders Møllekær, Rasmus Aagaard, Stefan Posth, Jesper Weile

**Affiliations:** 1 Research Unit at the Department of Respiratory Medicine, Odense University Hospital, Sdr. Boulevard 29, 5000 Odense C, Denmark; 2 Institute of Clinical Research, University of Southern Denmark, Odense, Denmark; 3 Department of Respiratory Medicine, Gentofte University Hospital, Gentofte, Denmark; 4 Department of Surgery, Odense University Hospital – Svendborg Hospital, Svendborg, Denmark; 5 Emergency Department, Odense University Hospital – Svendborg Hospital, Svendborg, Denmark; 6 Emergency Department, Holbaek Hospital, Holbaek, Denmark; 7 Department of clinical Medicine, Research Center for Emergency Medicine, Aarhus University, Aarhus, Denmark; 8 Department of Anesthesia, Randers Regional Hospital, Randers, Denmark; 9 Research Center for Emergency Medicine, Aarhus University, Aarhus, Denmark; 10 Emergency Department, Odense University Hospital, Odense, Denmark; 11 Research Center for Emergency Medicine, Aarhus University Hospital, Aarhus, Denmark; 12 Department of Emergency Medicine, Regional Hospital Herning, Herning, Denmark

## Abstract

The first Danish Society for Emergency Medicine (DASEM) recommendations for the use of clinical ultrasound in emergency departments has been made. The recommendations describes what DASEM believes as being current best practice for training, certification, maintenance of acquired competencies, quality assurance, collaboration and research in the field of clinical US used in an ED.

## Introduction

The purpose of these recommendations is to create a consensus for the use of clinical ultrasound (US) by an emergency department (ED) physician in the EDs in Denmark. The recommendations intend to describe the current best practice for training, certification, maintenance of acquired competencies, quality assurance, collaboration and research in the field of clinical US used in an ED.

The use of clinical US in emergency departments in Denmark is still early in its development. The scope of these recommendations is to set the organisational framework for how clinical US is implemented and used in the EDs in Denmark in the years to come. The scope of the recommendations is not to provide evidence-based guidelines on specific types of emergency medicine ultrasound. These are currently being established at an international level and the Danish Society for Emergency Medicine (DASEM) plan to endorse them, rather than creating alternative evidence based guidelines [1]. DASEM intends to inform and keep its members up-to-date of these guidelines at national meetings and by the use of the society’s webpage.

Highly specialised, diagnostic, US performed by specialists (e.g. radiologists, cardiologists) in an ED is beyond the scope of these recommendations, since these areas generally have their own guidelines and recommendations [2]. Clinical US is also an emergency medicine procedure and should not be considered in conflict with other US procedures performed by other specialists. Clinical US in the ED is used for bedside diagnosis, resuscitation, monitoring and procedural guidance of the critically ill or injured patient. US is performed, interpreted and integrated in a rapid manner directed by the clinical scenario [3].

### Recommendation panel

The DASEM committee on emergency medicine ultrasound established a panel consisting of representatives from each of the different health care regions in Denmark. At an initial meeting the overall content and scope of the recommendations was discussed and one of the panel members (CBL) subsequentially made an initial draft. This was then discussed and reviewed by all panel members at subsequent meetings and continues email correspondence. The final recommendations therefore represents a consensus paper in which representatives from each of the different geographical health care regions has agreed on an organisational setup of emergency medicine US which can be implemented in the ED’s in Denmark.

### Clinical ultrasound in emergency departments – scope of practice

In other recommendations emergency US has been classified into the following functional clinical categories [3]:

1. *Resuscitative*: Ultrasound use directly related to resuscitation

2. *Diagnostic*: Ultrasound utilised in an emergent diagnostic imaging capacity

3. *Symptom or sign-based*: Ultrasound used in a clinical pathway based upon the patient’s symptom or sign (e.g. trauma, red swollen leg)

4. *Procedure guidance*: Ultrasound used as an aid to guide a procedure

5. *Therapeutic and Monitoring*: Ultrasound use in therapeutics or in physiological monitoring

Various aspects of the above mentioned categories can also be described as focused ultrasonography or point-of-care ultrasonography (POCUS). The different EDs in Denmark have a very varied organisational set-up. Some patient categories which are routinely assessed in one ED may in other EDs be directly admitted to a highly specialised department. The access to highly specialised US examinations may also vary significantly. Therefore the different types of clinical US typically needed may vary from one ED another ED. The following types of POCUS can be seen as the basic curriculum which will be able to serve as the recommended POCUS “backbone” in most EDs:

1. *Focused Echocardiography* (e.g. Focus Assessed Transthoracic Echocardiography (FATE), Focused Echocardiography in Emergency Life Support (FEEL) [4,5])

2. *Focused Lung Ultrasound* (FLUS)

3. *Focused Abdominal Ultrasonography* (FAS)

4. *Extended Focused Assessment with Sonography for Trauma* (E-FAST)

5. *Focused Musculoskeletal Ultrasonography* (FMUS)

6. *Limited Compression Ultrasonography* (LCU)

The above mentioned types of POCUS should be used in accordance with international evidence based guidelines whenever such are available [1]. The DASEM committee on emergency medicine ultrasound intends to provide specific up-to-date descriptions on each of these types of POCUS, the purpose of the examinations as well as proposed standard reporting schemes at the society’s webpage.

The following types of US guided procedures are the recommended basic curriculum, which will enable the ED physician to use US for a vast range of common, acute invasive procedures in an ED:

– Vascular access

– US guided drainage/puncture of pleural effusion/pneumothorax

– US guided drainage/puncture of ascites

– US guided suprapubic placement of bladder catheter

– US guided nerve blocks

An ED physician using clinical US, should not necessarily be constrained by the above mentioned core curriculum. Other types of POCUS can be used as long as it is in accordance with the recommendations listed below. Research in clinical US in emergency departments is readily developing and the above mentioned core curriculum is expected to expand within the next couple of years.

### Training, certification and re-certification in clinical ultrasound for use in an ED

Education and training in clinical US should be based on the following elements

1. Theoretic basic knowledge

2. Practical hands-on training

3. Supervision of performed scans

4. Certification

5. Maintenance and further acquisition of US skills

6. Re-certification

Ideally, element 1 to 4 should be acquired as a formalised education endorsed by DASEM. The education can either be in a single type of clinical US examination (e.g. FAST) or several of the types mentioned in the curriculum. Each of the 6 educational elements is described in detail below.

### Theoretical basic knowledge

Basic knowledge of US physics, “knobology”, indications, contraindications, sonoanatomy, examination technique, pitfalls and limitations for the different types of sonography as well as correct storage and documentation of US images should be a part of the US curriculum. Traditionally many US guidelines typically describe how many hours of theoretical education is needed in order to reach a certain level of competencies, but as most theoretical education in Denmark is now being performed as e-learning courses, focus should be on course content rather than length. After completion of the theoretical training the trainee should be assessed with a test or exam in order to make sure that a sufficient level of knowledge has been obtained.

### Practical hands-on training

Hands-on training is mandatory and the course participant should be allowed as much time as possible for practical training in the different types and elements of clinical US. After completion of the practical training the trainee should be assessed with a practical test in order to make sure that a sufficient level of practical skills has been obtained making the trainee capable of performing the relevant type of clinical sonography under direct supervision.

### Supervision of performed scans

After completing theoretical and practical training, the trainee performs clinical sonography under direct supervision. Ideally this takes place in the ED where the trainee normally works. Currently in many EDs there is a lack of physicians with sufficient US skills to supervise other physicians in clinical US. Therefore educational clerkships in other departments in combination with web based supervision can serve as an alternative to supervision in the trainee’s own department. Prior to certification all examinations performed by the trainee should generally be reviewed.

For most types of POCUS examinations, the number of supervised scans needed in order to be able to perform POCUS at an adequate level is still unclear and may vary significantly from one trainee to another. In many types of POCUS 25 supervised scans have been used as a rule of thumb. Until further research in the field is available it is recommended that the novice should perform a minimum of 25 supervised scans for each type of examination. Each trainee should continually discuss with the mentor (see below) whether further scans are needed in order to obtain skills which are sufficient to be certified. All supervised examinations should be stored and documented in a logbook. Currently there is no official logbook, but the DASEM Committee on Clinical Ultrasound in Emergency Medicine aims to develop an official e-logbook for this purpose.

### Mentor

As a part of the educational program each trainee should have a mentor assigned. The mentor should help the trainee obtain the number of supervised examinations needed in order to become certified. The mentor should be certified in emergency medicine POCUS and be working at the same department as the trainee. Currently the number of ED physicians with sufficient US skills to function as a mentor is scarce. As an alternative, physicians with formalised training in diagnostic US can serve as mentors. If this is the case, the course faculty should ensure that the content of the educational program is clinical US for the use in an ED (e.g. POCUS) and not traditional diagnostic US.

### Certification

Theoretical skills, patient communication, practical skills, and knowledge on how to store and handle obtained US examinations should be assessed and certified. The purpose of certification is to ensure a uniform level of trainee competence no matter where or in which educational program the trainee is enrolled. Therefore every certification should be subject to external review. Censor(s) can either be representatives from DASEM or from one of the Danish university centres in clinical US.

### Maintenance of US skills

After completion of an educational program, the trainee should still keep a logbook and document the different types and number of examinations being performed. As part of a continuous quality management (see below), both logbook and a number of the recorded examinations should be subject to audit.

### Re-certification

Every 5^th^ year after completion of an educational program in clinical US for the use in an ED, the physician needs re-certification. Recertification should as a minimum include: Documentation of the number of US examinations performed (logbook), a one day course with brush up on theory and reassessment of both theoretical and practical skills. The individual physician is responsible for applying for a recertification and brush-up course and the course providers are responsible for providing such a course. As with the initial education, representatives from DASEM and/or one of the Danish university centres in clinical US must be present at the recertification course as censors.

### Collaboration with other institutions

Currently all universities in Denmark have begun including clinical US as a part of the pre-graduate curriculum of medical students. The pregraduate curriculums often contain elements of clinical US in the field of emergency medicine. DASEM encourages the making of collaborative networks between DASEM, the EDs and universities in Denmark. Such networks have potential for significantly improving quality of both pre- and postgraduate teaching in clinical US in the emergency medicine setting.

### DASEM endorsement of an educational program in clinical ultrasound for the use in EDs

In order to obtain DASEM endorsement, an educational program should be based on the above mentioned principles. The course program, portfolio and certification process must be approved by the DASEM Board and the DASEM Committee on Clinical Ultrasound in Emergency Medicine.

### Organisation of clinical ultrasound in an ED

**Figure 1:**
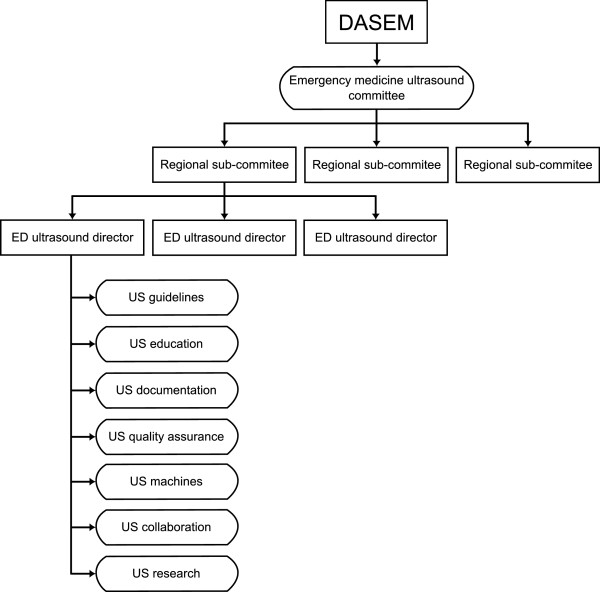
Organisation of emergency medicine ultrasound in Denmark.

In order to further establish and develop the use of clinical US in EDs in Denmark, an organisational structure within DASEM and the EDs is needed to promote the purpose. The recommended structure is illustrated in Figure 1.

### DASEM committee on clinical ultrasound in emergency medicine

The committee’s functions are:

– To advise the DASEM board and regional sub-committees in matters concerning the use of clinical US in emergency departments

– Production and maintenance of national guidelines in emergency medicine clinical US

– National coordination and monitoring of the education in emergency medicine clinical US

– National coordination and monitoring of US documentation

– National coordination and monitoring of quality assurance and management

– Collaboration with other medical societies and institutions

– National coordination and monitoring of research in the field of emergency medicine clinical US

### Regional sub-committee

The regional sub-committee’s functions are:

– To advise the regional emergency US directors in matters concerning the use of clinical US in emergency departments

– Regional coordination and monitoring of education in emergency medicine clinical US

– Regional coordination and monitoring of US documentation

– Regional coordination and monitoring of quality assurance and management

– Collaboration with other regional institutions and departments

– Regional coordination of research in the field of emergency medicine clinical US

### Emergency medicine clinical ultrasound director

Every ED should have an emergency medicine clinical ultrasound director. The director is a physician employed in the ED who has the administrative and educational oversight of the use of clinical US in the emergency department. The director must have obtained certification in US according to the DASEM guidelines in this paper or an equivalent US certification. The responsibilities of the director may vary from one ED to another, but typically they should consist of the following:

– Production and maintenance of department guidelines in clinical US

– Coordination of the departments education in emergency medicine clinical US

– US documentation

– Quality assurance and management

– Machine acquisition and maintenance

– Collaboration with other departments and institutions

– Coordination of research

– Creation of US reporting schemes for use in the patient’s electronic medical chart

### Guidelines

Specific guidelines describing the departments US education, certification, machine maintenance, quality assurance and management should be available at every emergency medicine department.

### Educational guidelines

The overall education in clinical US for the use in an emergency department has been described in detail above. Beside this every ED is further encouraged to make specific guidelines on:

– How to achieve a sufficient number of supervised US examinations in order to be able to complete certification

– How to maintain and document US skills

– How to ensure that re-certification is completed

### Documentation

The use of standardised documentation and storage of acquired US images and movie clips has traditionally been limited to US examinations performed by specialists. In the ED setting this can be a challenging task, since most US examinations are focused, goal-directed and performed and interpreted at the bedside. DASEM recommends standardised storage of US images/movie clips at every US examination performed in an ED, though, storage should not be at the expense of a delay in any acute treatment of the patient. A systematic storage of US examinations has several advantages: Documentation of positive and negative findings, older images can more easily be acquired for comparison, review of stored examinations can be performed as part of quality assurance (QA), stored examinations can be used for educational purposes, and findings can be presented when assistance from other collaborators is needed. Most hospitals in Denmark have a database system for storing US examinations. The EDs ought to have access to such a system. Further, all US examinations should be described and documented in the patient’s electronic journal. The DASEM committee on emergency medicine ultrasound intends to provide standard reporting schemes for each type of POCUS at the society’s webpage.

In order to facilitate documentation as a standardised part of any US examination in an ED, storage and documentation should be included as part of the curriculum of US training.

### Quality assurance of an ED physician performing clinical ultrasound

Quality assurance (QA) should be based on direct observation and audit of stored examinations and image interpretation. The purposes of the QA of an ED physician performing clinical US are:

– Evaluation of technical aspects of clinical US (e.g. image quality, correct use of presets)

– Evaluation of interpretation and clinical accuracy

– Provide feedback

– Monitoring whether the use of clinical US is performed in accordance with guidelines and best current practise.

Audit and QA should be seen as integrated parts of the everyday use of clinical US in an ED. An example of how an audit of examinations can be performed is (adapted from the ACEP (American College of Emergency Physicians) recommendations):

1. Images and video clips obtained by the physician are exported to scanner hard drive and exported to an external archive

2. The physician documents US findings and interpretation in the electronic patient journal

3. The stored images or clips and electronic medical chart documentation are then reviewed on a sample basis by the US director or his/her designee

4. Reviewers evaluate images for technical quality and interpretation and submit the result of the audit back to the physician

5. The result of the audit is archived and available for review later should they be needed

### Quality assurance of an ED where physicians perform clinical ultrasound

The purposes of the QA of an ED where physicians perform clinical US are:

– Overall evaluation of the departments technical aspects of clinical US (e.g. image quality, correct use of preset)

– Overall evaluation of the departments interpretation and clinical accuracy

– Provide feedback to the department and emergency medicine clinical US director

– Monitoring whether the use of clinical US in the ED department is performed in accordance with guidelines and best current practise.

– Monitoring of cases in which the “border-zone” between POCUS in an ED and the use of diagnostic US in a highly specialised department could or did compromise patient safety

The emergency medicine clinical US director is responsible for the overall quality assurance of an ED where physicians perform clinical US. This should comprise local, institutional, and national aspects.

At the institutional level, the US director should facilitate collaboration with specialised departments using diagnostic US.

At the national level, as part of the DASEM organisation, US directors from the EDs in Denmark should have regular meetings with representatives from the DASEM Committee on Clinical Ultrasound in Emergency Medicine. The DASEM committee on clinical ultrasound will be responsible for auditing EDs on a 5-year cycle to ensure the ultrasound director is keeping good records and ensuring compliance with these guidelines.

### Ultrasound machines

Every ED in Denmark should have dedicated US machines located in the ED for use by emergency physicians. The ED should have guidelines describing: Regular in-service of personnel using the equipment and appropriate transducer care, stocking and storage of supplies, adequate cleaning of transducers, upkeep and maintenance of US machines by medico technical staff.

### Use of more advanced forms of diagnostic ultrasound in an ED

Several societies have specific recommendations for the use of diagnostic US in a variety of medical specialities. Currently emergency medicine is not a formal speciality in Denmark; hence physicians working in the EDs have very different backgrounds and may be educated and certified in diagnostic US within a speciality (e.g. diagnostic echocardiography). As long as the physicians’ education, certification and maintenance of acquired competencies are in accordance with recommendations from national and international societies, there is no preclusion for the physicians to perform more advanced forms of diagnostic US in the ED.

### Research

DASEM wants to promote cutting edge research activities within the field of emergency medicine clinical US. The overall research strategy should focus on developing evidence based guidelines. Research should include both individual and multicentre studies investigating different aspects of emergency medicine clinical US.

## Conclusion

The use of clinical US in the EDs in Denmark is still early in its development. Hopefully these recommendations will help set the framework for implementation and use of clinical US in the EDs in Denmark in the years to come. A revised edition of these DASEM recommendations is expected to be published in 2018. The revised edition intends to incorporate any future recommendations set forth by the International Federation for Emergency Medicine.

## Competing interests

The authors declare that they have no competing interest.

## Authors’ contributions

All authors have contributed to conception and design, acquisition, review, and interpretation of current literature. At an initial meeting the overall content of the manuscript was discussed by all authors. The first author of the manuscript subsequentially made an initial draft which was discussed and reviewed by the working group at subsequent meetings and email correspondence. All authors have approved the final version to be published.
